# Influenza vaccination coverage and factors associated with severe laboratory-confirmed influenza-related illness in patients receiving care at a tertiary hospital in Catalonia (Spain) during the 2018–2019 epidemic season

**DOI:** 10.1371/journal.pone.0260397

**Published:** 2021-12-02

**Authors:** Guillermo Mena, Irma Casas, Cristina Casañ, Mario Auñón, Lurdes Matas, Josep-Maria Mòdol, María Esteve

**Affiliations:** 1 Servicio de Medicina Preventiva, Hospital Universitari Germans Trias i Pujol, Badalona, Spain; 2 Universitat Autònoma de Barcelona, Barcelona, Spain; 3 Servicio de Microbiologia, Laboratori Clínic Metropolitana Nord, Hospital Universitari Germans Trias i Pujol, Badalona, Spain; 4 CIBER Epidemiology and Public Health (CIBERESP), Instituto de Salud Carlos III, Madrid, Spain; 5 Dirección Médica, Hospital Universitari Germans Trias i Pujol, Badalona, Spain; Johns Hopkins University, UNITED STATES

## Abstract

**Introduction:**

Influenza vaccination rates in risk groups remain suboptimal. Evidence supporting a significant association between influenza vaccination and severe illness is limited.

**Methods:**

We retrospectively analyzed the epidemiological characteristics of out- and inpatients with laboratory-confirmed influenza infection attended during the 2018–19 epidemic season. Influenza vaccination coverage by indication was analyzed. Logistic regression was used to compare the odds of vaccination between severe and non-severe influenza-positive patients. Severe cases were defined as presenting pneumonia, admission to critical care units and/or death.

**Results:**

The overall vaccination coverage among influenza-positive patients was 30.4%. In subjects with ≥ 1 indication for vaccination, the vaccination coverage was 42.4%. By indication, coverage rates were: 52.5% in patients aged ≥ 59 years, 42.2% in obese patients, 29.2% in immunosuppressed subjects and 6.5% in pregnant women. In patients with underlying chronic diseases, a higher coverage was found in patients with cognitive impairment (77%), muscular dystrophy (63.6%) and renal disease (60.4%). The multivariate logistic regression model showed severe influenza-related illness was associated with a lack of influenza vaccination before seeking care during the 2018–2019 season [0.59 (95%CI 0.36–0.97); p = 0.038], older age [1.01 (95%CI 1.00–1.02); p = 0.009] and current or former smoking status [1.63 (95%CI 0.84–3.18) and 2.03 (95%CI 1.16–3.57); p = 0.031], adjusted by underlying disease.

**Conclusion:**

Adjusting by age, smoking status and underlying disease, a moderate association between the influenza vaccine and severe laboratory-confirmed influenza-related illness was found in an epidemic season in which there was matching between the vaccine and circulating strains. Protection against complications, especially in older subjects and in those with underlying disease is postulated as one of the strengths of annual influenza vaccination. However, influenza vaccination is a pending issue in these groups, especially in pregnant women and obese people. To avoid suboptimal vaccination coverages, health professionals should recommend the seasonal influenza vaccination according to the annual instructions of the health authorities.

## Introduction

According to the US Centers for Disease Control and Prevention, the World Health Organization and global health partners, annual influenza epidemics affect 5–15% of people worldwide, with as many as 650,000 influenza-related deaths [1].

Influenza symptoms vary widely from mild to lower respiratory tract infections, leading to respiratory and multi-organ failure and death. Extra-pulmonary complications, such as viral myocarditis and encephalitis have also been described [2]. Influenza may affect people of any age, but complications and hospitalizations are more frequent in childhood, especially in children aged <5 years, pregnant women, and people with obesity, chronic disease, and older age [3–5].

Influenza vaccination is recommended for people aged ≥ 60 years, those with underlying health conditions, pregnant women, and healthcare workers, among others. The match between circulating and vaccine strains determines the vaccine effectiveness for each season. Although the post-infection effects of vaccination must be also taken into consideration, evidence supporting a positive effect of influenza vaccination in the prevention of severe illness is limited [6, 7].

The surveillance of severe hospitalized cases of confirmed influenza (SHLCI) was introduced a decade ago by the Catalan Public Health Agency within the influenza sentinel surveillance system (PIDIRAC) [8, 9]. The preventive medicine department of Germans Trias i Pujol Hospital (GTiPH) in Badalona (Spain) notifies seasonal data to the SHLCI including influenza-related pneumonia, intensive care unit admissions and deaths in patients admitted to hospital for laboratory-confirmed influenza. The data includes information on hospital admissions, the clinical presentation, underlying diseases, vaccination, antiviral treatment, and deaths [9, 10].

The objectives of this study were to describe the epidemiological and clinical characteristics of all-age laboratory-confirmed influenza cases, to estimate influenza vaccination coverages in high risk patients, and to evaluate factors associated with severe influenza-related cases (pneumonia, admission to critical care units and/or deaths) in patients receiving care at a tertiary Spanish hospital during the 2018–2019 epidemic season.

## Material and methods

### Study design

The study was performed at Germans Trias University Hospital, which is a reference center for 800,000 people in Catalonia, Spain. We retrospectively analyzed the epidemiological characteristics of out- and inpatients with laboratory-confirmed influenza infection attended during the 2018–19 epidemic season.

The study included all laboratory-confirmed influenza cases from epidemiological week (EW) 40/2018 to 20/2019. Information on age, sex, underlying comorbidities, smoking, admission and discharge dates, severe illness (pneumonia, intensive care unit admission and/or death) and type of influenza virus (A, H1N1, H3N2 or B) was obtained from the electronic medical record. The influenza vaccination status was obtained from the regional vaccination register. Subjects were considered protected 14 days after vaccine administration.

### Laboratory analysis

Laboratory data were obtained from routine testing procedures, as described below. Samples were obtained through throat and nasopharyngeal swabs and collected with flocked swabs in viral universal transport medium (Deltalab S.L, Barcelona, Spain); nasal wash/aspirate specimens were collected in clear polypropylene containers. Specimens were received at the laboratory within 30 minutes after collection and were stored at 4°C until processed, always within 12h after reception. Leftover positive samples were conserved at -80°C.

Two real-time PCR for influenza A/B and VRS detection were used: AllplexTM Respiratory Panel 1 (Seegene, Seoul, Korea) and Cobas Liat Inf A/B (Roche Molecular System, Branchburg, NJ, USA). AllplexTM Respiratory Panel is a qualitative multiplex real-time PCR assay that detects and identifies influenza types A & B, respiratory syncytial virus types A y B, and influenza A subtypes H1, H3 and H1pdm09. Cobas Liat is a rapid, real-time RT-PCR test for the qualitative detection of nucleic acid from influenza A/B. The Allplex assay requires nucleic acid extraction, which was performed using the Nimbus platform (Hamilton Company, Reno, US) following a protocol established according to the manufacturer’s instructions. The real-time PCR thermocycler used for amplification was CFX96™ Real-Time PCR Detection System (Bio-Rad). The software designed by each company was used for detection and data analysis.

### Influenza vaccination recommendations

People aged ≥ 60 years, pregnant women and patients with underlying health conditions are offered influenza vaccination free of charge each season in Spain. During the 2018–2019 season, subjects aged ≥ 65 years received the Chiromas® vaccine [11], a trivalent inactivated influenza vaccine manufactured by Seqirus, which contains the MF59 adjuvant. The Fluarix Quadrivalent® vaccine [12] was mainly used in immunosuppressed persons aged ≥ 6 months. Chiroflu® [13], a non-adjuvanted trivalent vaccine was administered to persons not included as targets for the first two vaccines.

### Statistical analysis

A descriptive analysis was made of all patients attended (out- and in- patients) with laboratory-confirmed influenza during the 2018–2019 season. The influenza vaccination coverage was expressed as the quotient between vaccinated patients during the 2018–2019 and patients testing positive for influenza. The characteristics of severe and non-severe influenza inpatients were compared using the Chi-square test or Fisher’s exact test for categorical variables, and the Student’s t-test test for continuous variables. P-values <0.05 were considered statistically significant. Logistic regression was used to compare the odds of vaccination between severe and non-severe influenza-positive patients. Crude and adjusted odds ratios (OR) and their 95% confidence intervals (CI) were calculated. All data were analyzed using Excel (Microsoft, Redmond, WA, US) and IBM SPSS Statistics for Windows, version 24 (IBM Corp., Armonk, NY, USA).

### Ethical considerations

The planning, conduct and reporting of the study were in line with the Declaration of Helsinki World Medical Association [14]. Data were collected as part of in-hospital surveillance activity during the 2018–2019 influenza season. The requirement to obtain informed consent was waived because nasopharyngeal swab sampling was performed for surveillance purposes and considered a noninvasive intervention. The data collected were encoded for the researchers. The research protocol was approved by the GTiPUH Ethics Committee (ref: INFLUSEVERITY_19).

## Results

**Fig 1:**
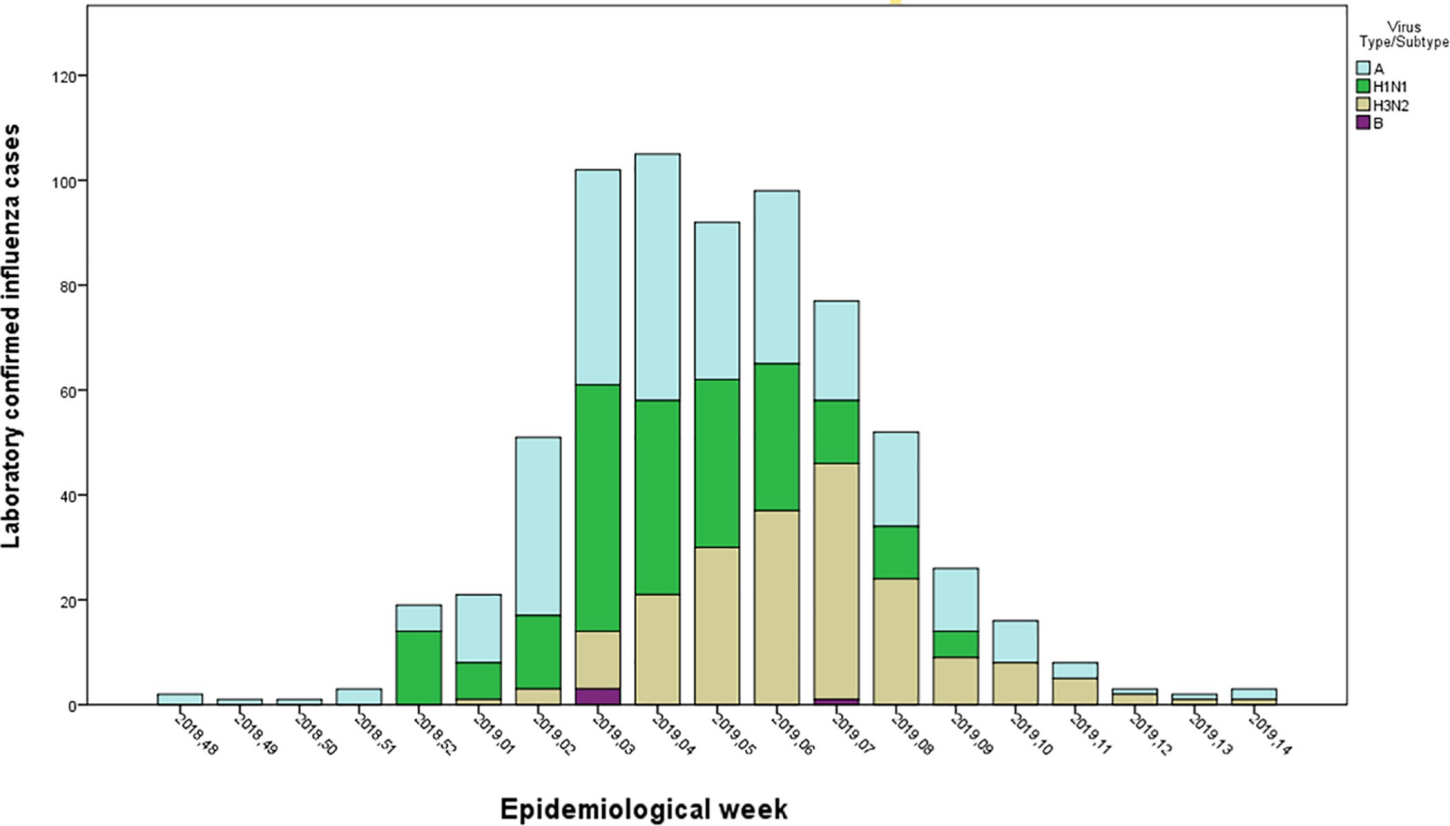
Influenza virus types/subtypes detected by epidemiological week at the GTiPUH laboratory during the 2018–2019 influenza season.

During the 2018–2019 epidemic season, 682 confirmed cases of influenza virus infection were identified. The first and last cases were detected in EW 48/2018 and 14/2019, respectively. Of the 678 influenza virus type A positive samples, 274 (40.2%) were not subtyped, 198 (29%) were A(H1N1)pdm09, and 206 (30.2%) were A(H3N2). The A(H1N1)pdm09 and A(H3N2) subtypes co-circulated during the epidemic season. The A(H1N1)pdm09 strain was more prevalent until EW 06/2019. From then until the end of the epidemic season, A(H3N2) was the most frequently detected subtype (Fig 1).

Of the cases analyzed, 48.5% were male. The median age was 55 years (IQR: 13–75) and 37.5% of patients were aged ≥ 65 years, 19.8% 45–64 years and 18.5% 0–4 years. Thirty-one pregnant women tested positive for influenza virus. Never-smokers accounted for 78.8% of patients and 64.4% had ≥ 1 underlying chronic disease (Table 1).

**Table 1:** Baseline characteristics of laboratory-confirmed influenza attended during the 2018–2019 influenza season (n = 682).

	Laboratory–confirmed cases^#^ (n = 682)	Outpatients (n = 394)	Inpatients (n = 288)			
			Overall (n = 288)	Patients with pneumonia (n = 95)	ICU admissions (n = 18)	Patients with severe illness* (n = 113)
**Sex, n (% male)**	331 (48.5)	179 (45.4)	152 (52.8)	46 (48.4)	6 (33,3)	55 (48.7)
**Age in years, median (IQR)**	55 (13–75)	37.5 (5–63)	69 (48–81)	63 (39–80)	59 (46–71.5)	63 (42–79.5)
**Age, n (%)** **						
0-4y	126 (18.5)	96 (24.4)	30 (10.4)	13 (13.7)	0	13 (11.5)
5-14y	47 (6.9)	39 (9.9)	8 (2.8)	2 (2.1)	0	2 (1.8)
15-44y	118 (17.3)	93 (23.6)	25 (8.7)	12 (12.6)	4 (22.2)	14 (12.4)
45-64y	135 (19.8)	73 (18.5)	62 (21.5)	22 (23.2)	7 (38.9)	28 (24.8)
65+y	256 (37.5)	93 (23.6)	163 (56.6)	46 (48.4)	7 (38.9)	56 (49.6)
**2018–2019 vaccination status, n (%vaccinated)**	206 (30.4)	86 (21.9)	120 (42.1)	24 (25.5)	2 (11.1)	32 (28.8)
**Influenza virus infection, n (%)**						
A (Untyped)	274 (40.2)	192 (48.7)	82 (28.5)	27 (28.4)	3 (16.7)	31 (27.4)
A(H1N1)pdm09	206 (30.2)	103 (26.1)	103 (35.8)	42 (44.2)	10 (55.6)	49 (43.4)
H3N2	198 (29)	96 (24.4)	102 (35.4)	26 (27.4)	5 (27.8)	33 (29.2)
B (Untyped)	4 (0.6)	3 (0.8)	1 (0.3)	0	0	0
**Immunosuppressed, n (%)**	107 (15.7)	63 (16)	44 (15.3)	20 (21.1)	5 (27.8)	22 (19.5)
**Pregnant, n (%)**	31 (4.5)	27 (6.9)	4 (1.4)	2 (2.1)	0	2 (1.8)
**Obesity, n (%)**	44 (6.5)	16 (4.1)	28 (9.7)	6 (6.3)	3 (16.7)	9 (8)
**Underlying disease, n (%)** ***	439 (64.4)	206 (52.3)	233 (80.9)	68 (71.6)	16 (88.9)	85 (75.2)
**Hospital stay in days, median (IQR)**	0 (0–5)	-	7 (3–12)	6 (2–10.5)	11.5 (7–17)	7 (3–14)
**Smoking status, n (%)**						
Non-smoker	533 (78.8)	338 (86.7)	195 (68.2)	66 (89.5)	7 (38.9)	74 (65.5)
Former smoker	85 (12.6)	25 (6.4)	60 (21)	21 (22.1)	4 (22.2)	25 (22.1)
Smoker	58 (8.6)	27 (6.9)	31 (10.8)	8 (8.4)	7 (38.9)	14 (12.2)
**Days between symptom onset and positive swab, median (IQR)**	3 (1–6)	3 (1–6)	3 (1–6)	5 (2–7)	5 (3–7.25)	5 (2–7)

^**#**^ Laboratory-confirmed cases includes out- and inpatients.

Severe illness: pneumonia, intensive care unit admission and/or death. Sixteen patients died during hospitalization.

**Age categories: according to PIDIRAC (Surveillance of Seasonal Influenza *in Catalonia)*^*2*^.

ICU: Intensive Care Unit.

***Underlying disease: Diabetes mellitus, obesity, cardiovascular disease, respiratory disease, renal disease, liver disease, muscular dystrophy, cognitive impairment, active cancer and/or immunosuppression.

The overall vaccination coverage was 30.4%. In subjects with ≥ 1 indication for vaccination, the vaccination coverage was 42.4% (n = 486) (Table 2). By indication, coverage rates were: 52.5% (n = 307) in patients aged ≥ 59 years, 6.5% (n = 31) in pregnant women, 29.2% (n = 107) in immunosuppressed subjects and 42.2% (n = 44) in obese patients. In patients with underlying chronic diseases, a higher coverage was found in those with cognitive impairment (77%, n = 61), muscular dystrophy (63.6%, n = 11), renal disease (60.4%, n = 73), cardiovascular disease (56.2%, n = 121), diabetes mellitus [DM] (53.8%, n = 119) and respiratory disease (47%, n = 185). In patients with ≥ 2 underlying chronic diseases, the vaccination coverage was higher, especially in those with cognitive impairment and cardiovascular disease (91.7%, n = 12), DM (90.9%, n = 11) and respiratory disease (69.2%, n = 13), and in patients with renal and cardiovascular (80.6%, n = 31) or respiratory disease (63.8%, n = 22), and when DM was combined with renal disease (69.7%, n = 33), cardiovascular disease (64.3%, n = 42) and respiratory disease (61.7%, n = 47). A lower coverage was observed in patients with liver disease (44.2%, n = 43), even when combined with respiratory disease (20%, n = 5).

**Table 2:** Percentage of influenza-vaccinated patients by underlying conditions in laboratory-confirmed influenza cases receiving care at the GTiPUH in Badalona (Spain) during the 2018–2019 influenza epidemic season.

	Diabetes Mellitus	Muscular dystrophy	Cognitive impairment	Renal disease	Cardiovascular disease	Respiratory disease	Immuno-suppression	Liver disease	Obesity	Pregnancy	≥60 years	≥65 years
	n = 119 (53.8%)	n = 11 (63.6%)	n = 61 (77%)	n = 73 (60.4%)	n = 121 (56.2%)	n = 185 (47%)	n = 106 (29.2%)	n = 11 (27.3%)	n = 43 (44.2%)	n = 31 (6.5%)	n = 307 (52.5%)	n = 253 (57.3%)
Diabetes mellitus		n = 3 (66.7%)	n = 11 (90.9%)	n = 33 (69.7%)	n = 42 (64.3%)	n = 47 (61.7%)	n = 23 (30.4%)	n = 1 (0%)	n = 14 (57.1%)	n = 0	n = 100 (56%)	n = 88 (60.2%)
Muscular dystrophy			n = 7 (85.7%)	n = 1 (100%)	n = 2 (50%)	n = 1 (0%)	n = 1 (0%)	n = 0	n = 1 (100%)	n = 0	n = 1 (100%)	n = 0
Cognitive impairment				n = 8 (50%)	n = 12 (91.7%)	n = 13 (69.2%)	n = 3 (66.7%)	n = 0	n = 4 (75%)	n = 0	n = 41 (78%)	n = 35 (80%)
Renal disease					n = 31 (80.6%)	n = 22 (63.8%)	n = 13 (53.8%)	n = 3 (33.1%)	n = 6 (66.7%)	n = 0	n = 61 (60.7%)	n = 54 (64.8%)
Cardiovascular disease						n = 47 (59.6%)	n = 12 (66.7%)	n = 4 (25%)	n = 17 (58.8%)	n = 0	n = 111 (57.7%)	n = 99 (59.6%)
Respiratory disease							n = 20 (45%)	n = 5 (20%)	n = 23 (47.8%)	n = 7 (0%)	n = 128 (59.4%)	n = 109 (62.4%)
Immunosuppression								n = 1 (0%)	n = 1 (0%)	n = 0	n = 54 (40.7%)	n = 41 (46.3%)
Liver disease									n = 0	n = 0	n = 8 (25%)	n = 7 (28.7%)
Obesity										n = 0	n = 29 (48.3%)	n = 22 (50%)

With respect to influenza-associated complications, 95 (13.9%) influenza-positive cases presented pneumonia before or during hospitalization. Eighteen (2.6%) patients were admitted to an ICU and 16 (2.3%) died. The median age in patients admitted to the ICU was 59 years (IQR:46–71.5). No child was admitted to the ICU. Of the 18 patients admitted to the ICU, 16 (88.9%) presented comorbidities. There were no pregnant women among the ICU patients. Seventeen of these patients (94.4%) were candidates for vaccination before hospitalization but only two were vaccinated.

The univariate analysis of patients with and without severe influenza-related illness is shown in Table 3.

**Table 3:** Univariate analysis of factors associated with severe influenza-associated illness.

	Patients without severe influenza related illness (n = 569)	Patients with severe influenza-related illness * (n = 113)	p
**Sex, n (% male)**	276 (48.5)	55 (48.7)	0.974
**Age in years, median (IQR)**	50 (8–74)	63 (42–79.5)	**<0.001**
**Age > = 65 yrs. n (%)**	200 (35.1)	56 (49.6)	**0.004**
**Smoking status, n (%)**			**<0.001**
Non-smoker	459 (81.5)	74 (65.5)	
Former smoker	60 (10.7)	25 (22.1)	
Smoker	44 (7.8)	14 (12.2)	
**A(H1N1)pdm09 infection** ** **, n (%)**	157 (48.8)	49 (58.8)	**<0.001**
**Underlying disease** ***, n (%)	354 (62.2)	85 (75.1)	**0.008**
**Obesity, n (%)**	35 (6.2)	9 (8)	0.474
**Immunosuppression, n (%)**	85 (14.9)	22 (19.5)	0.226
**Vaccinated, n (%)**	174 (30.7)	32 (28.8)	0.689

* Severe illness: pneumonia, intensive care unit admission and/or death.

**A(H1N1)pdm09 vs. H3N2 infection. Non-subtyped Influenza A infections were not included.

***Underlying disease: Diabetes mellitus, obesity, cardiovascular disease, respiratory disease, renal disease, liver disease, muscular dystrophy, cognitive impairment, active cancer and/or immunosuppression.

The bivariate analysis showed that older age [1.01 (95%CI 1.01–1.02); p <0.001], age ≥ 65 years [1.81 (95%CI 1.21–2.72); p = 0.004], smoker or former smoker status [1.97 (95%CI 1.03–3.78) and 2.58 (95%CI 1.53–4.38), respectively; p = 0.001], and any underlying disease [1.84 (95%CI 1.17–2.92); p = 0.009], were all associated with an increased probability of severe influenza-related illness (Table 4). Sex, type of influenza A virus, obesity, immunosuppression, and influenza vaccination as an unadjusted variable showed no significant association with severe influenza-associated illness.

**Table 4:** Bivariate and multivariate analysis of factors associated with severe influenza-associated illness.

	Bivariate analysis		Multivariate analysis	
	OR (CI)	*p*-Value	OR (CI)	*p*-Value
**Sex (male)**	0.99 (0.66–1.49)	0.974		
**Age in years**	1.01 (1.01–1.02)	**<0.001**	**1.01 (1.00–1.02)** ^	**0.009**
**Age > = 65 yrs.**	1.81 (1.21–2.72)	**0.004**		
**Smoking status**		**0.001**		**0.031**
Non-smoker	Ref.		Ref	
Smoker	1.97 (1.03–3.78)		1.63 (0.84–3.18)	
Former smoker	2.58 (1.53–4.38)		2.03 (1.16–3.57)	
**A(H1N1)pdm09 infection** ***	1.56 (0.95–2.56)	0.077		
**Underlying disease** ****	1.84 (1.17–2.92)	**0.009**	1.04 (0.56–1.91)	0.888
**Obesity**	1.32 (0.62–2.83)	0.475		
**Immunosuppression**	1.38 (0.82–2.31)	0.228		
**Vaccinated**	0.91 (0.58–1.43)	0.689	0.59 (0.36–0.97)	**0.038**

* Severe illness: pneumonia, intensive care unit admission and/or death.

**One case of influenza B virus was detected during the 2018–19 season, which was not severe.

***A(H1N1)pdm09 vs. H3N2 infection. Non-subtyped influenza A infections were not included.

****Underlying disease: Diabetes mellitus, obesity, cardiovascular disease, respiratory disease, renal disease, liver disease, muscular dystrophy, cognitive impairment, active cancer and/or immunosuppression.

^1.014 (1.003–1.024).

The multivariate logistic regression model showed that severe influenza-related illness was associated with a lack of influenza vaccination before the hospital visit during the 2018–2019 season [0.59 (95%CI 0.36–0.97); p = 0.038], older age [1.01 (95%CI 1.00–1.02); p = 0.009] and current or former smoking status [1.63 (95%CI 0.84–3.18) and 2.03 (95%CI 1.16–3.57); p = 0.031], adjusted by underlying disease.

## Discussion

The vaccination coverage in confirmed influenza-virus patients with ≥ 1 indication for vaccination was suboptimal (42.4%). We found a significant association between the lack of influenza vaccination, adjusted by age, smoking status and underlying disease, and severe influenza-related cases during the 2018–2019 epidemic, a season when circulation of the type A virus was predominant, and influenza B was marginal.

In terms of age, vaccination coverage was 57.3% in individuals aged ≥ 65 years. Cognitive impairment was the underlying factor independently associated with a very good vaccination coverage (77%); even more so when age ≥ 65 years (80%), muscular dystrophy (85.7%), DM (90.9%) or cardiovascular disease (91.7%) were added. The Spanish Health Ministry coverage objective of 65% was achieved only by summing two or more underlying conditions: muscular dystrophy plus DM (90.9%), renal disease plus cardiovascular disease (80.6%), renal disease plus DM (69.7%), immunosuppression plus cardiovascular disease (66.7%) and obesity plus renal disease (66.7%). No combination of age > 60 years and another underlying disease, except for cognitive impairment, reached the 65% coverage objective. Pregnant women had an extremely low influenza vaccination coverage (6.5%), and the coverage in persons with liver disease (27.3%), immunosuppression (29.2%), and obesity (44.2%) also showed wide room for improvement. In Spain, the Ministry of Health’s objective for the 2018–2019 season was to reach 65% coverage in persons aged ≥ 65 years and those with underlying diseases. However, during the season, influenza vaccination coverage in Spain reached 54.3% in persons aged ≥ 65 years and 40.6% in pregnant women (there are no national coverage data for the other indications) [15]. The World Health Organization aims to achieve a vaccination coverage of 75% in people aged ≥ 65 years and other high-risk population groups [16].

In Catalonia, there was an atypical circulation pattern, with initial AH1N1pdm09 virus circulation followed by co-circulation with the AH3N2 virus during the 2018–2019 epidemic season. In Catalonia, a large number of severe cases were hospitalized, but there was a lower incidence and case fatality rate compared with the 2017–2018 season [8]. In the 2017–2018 season, the GTiPUH laboratory detected 706 positive samples for influenza virus: Forty-three were admitted to the ICU (6.1%), and 26 died during hospitalization (3.7%). During the 2018–2019 season, of the 682 laboratory-confirmed cases, 18 patients (2.6%) were admitted to the ICU and 16 died (2.3%) [10]. Therefore, the GTiPUH laboratory trend showed a similar picture to that given by the PIDIRAC for all Catalonia.

The effectiveness of influenza vaccination in preventing influenza infection during the 2018–2019 season was measured in Spain. In Navarra, Castilla et al. estimated a vaccine effectiveness against influenza A(H1N1)pdm09 of 57% (95%CI 40 to 70%) and 12% (95%CI −23 to 37%) against influenza A(H3N2), using a test-negative design [17]. A mid-season test-negative analysis in Alicante showed an overall vaccine effectiveness in preventing influenza cases of 42.5% (95%CI -17 to 72)% [18]. We found a significant association between the lack of influenza vaccination and severe influenza-related cases during the 2018–2019 epidemic season. According to the phylogenetic analysis of influenza viruses during the season, most of influenza A genetic, including H1N1pdm09 and H3N2, were contained in the 2018–2019 season influenza vaccine [8].

Between-season comparisons of 6 influenza seasons in Catalonia (2010–2011 to 2015–2016) showed the adjusted effectiveness of influenza vaccination in preventing ICU admission or death was 22% (95%CI 1 to 39%) [3] and that some mismatching seasons (2014–2015 for influenza A virus and seasons 2011–2012, 2013–2014 and 2015–2016 for influenza B virus) may have influenced the effectiveness of the influenza vaccine. GTiPUH evaluation of the mismatched 2017–2018 season found influenza vaccination protected against severity (p = 0.004) after adjustment for age, sex, type of virus and complexity indices [10].

Multivariate regression showed severe influenza-related illness was associated with the lack of vaccination in the 2018–2019 season and with older age and current or former smoking, similar to the results of other studies. Older age is a known risk factor for hospital admission due to influenza, and age ≥ 65 years is a frequent cutoff for the risk of severe influenza and evaluation of vaccine effectiveness [19, 20]. Smoking increases the risk of hospital admission [21] and is a risk factor for influenza severity and the most common complications. However, associated comorbidities, including chronic obstructive pulmonary disease and congestive heart disease, mean it is difficult to evaluate the role smoking plays in increasing influenza severity [21, 22].

The study’s main strength is that GTiPUH surveillance comprehensively reviews the medical records of confirmed cases. The main limitation is that some variables that it would have been of interest to include in the multivariate analysis, including vaccination in preceding seasons, which has been associated with current vaccine effectiveness, [17] were not collected. Likewise, vaccine effectiveness could not be evaluated as no test-negative design was used. Finally, comparability of vaccination coverage is limited, since the proportion of vaccinated subjects was calculated in influenza-infected patients.

In conclusion, we found that after adjustment for independent factors such as age and smoking, there was a moderate association between influenza vaccination and severe laboratory-confirmed influenza-related illness in an epidemic season when the vaccine and circulating strains matched. Even though influenza vaccination provides protection against complications, especially in older people and those with underlying disease, the uptake in these groups remains low, particularly in pregnant women and obese people. Health professionals should recommend seasonal influenza vaccination according to the annual recommendations to avoid suboptimal coverages.
