# Correction: Influenza vaccination coverage and factors associated with severe laboratory-confirmed influenza-related illness in patients receiving care at a tertiary hospital in Catalonia (Spain) during the 2018–2019 epidemic season

**DOI:** 10.1371/journal.pone.0295454

**Published:** 2023-11-30

**Authors:** Guillermo Mena, Irma Casas, Cristina Casañ, Mario Auñón, Lurdes Matas, Josep-Maria Mòdol, María Esteve

There is a mistake in the Results section. This sentence is incorrect: “in patients aged ≥ 59 years”. The correct sentence is: “**in patients aged ≥ 60 years**”.

There are several mistakes in the Discussion section.

This sentence is incorrect: “No combination of age > 60 years and another underlying disease” The correct sentence is: “**No combination of age ≥ 60 years and any underlying disease**”.

This sentence is incorrect: “However, associated comorbidities, including chronic obstructive pulmonary disease and congestive heart disease, mean it is difficult to evaluate the role smoking plays in increasing influenza severity [21, 22].” The correct sentence is: “**However, the underlying comorbidities associated with smoking, such as chronic obstructive pulmonary disease and congestive heart disease, make it difficult to evaluate the role that smoking plays in increasing influenza severity [21, 22].**”

This sentence is incorrect: “Even though influenza vaccination provides protection against complications, especially in older people and those with underlying disease, the uptake in these groups remains low, particularly in pregnant women and obese people” The correct sentence is: “**Even though influenza vaccination provides protection against complications, especially in older people and those with health conditions, the uptake in these groups remains low, particularly in pregnant women and obese people.**”
